# Supplementary material for: Cerebrovascular Events after Transcatheter Aortic Valve Replacement: The Difficulty in Predicting the Unpredictable
Source: J Clin Med. 2022 Jul 4;11(13):3902. doi: 10.3390/jcm11133902 (PMC9267500; doi:10.3390/jcm11133902)
Supplement: Supplementary file 1 [file jcm-11-03902-s001.zip › jcm-1742461-supplementary.pdf]

Journal of Clinical Medicine

MDPI

Supplementary Materials

Figure S1. Modified CONSORT diagram of the derivation cohort.

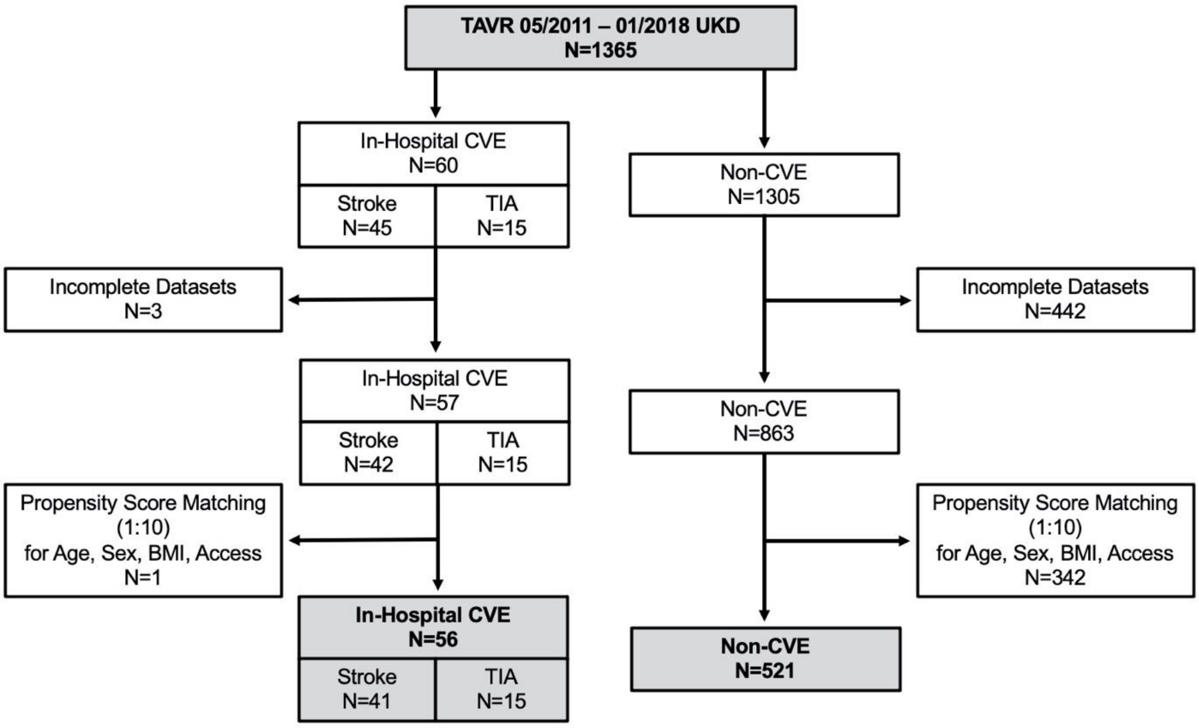

BMI = Body mass index, CVE = Cerebrovascular event, TAVR = Transcatheter aortic valve replacement, TIA = Transient ischemic attack.

Figure S2. Modified CONSORT diagram of the validation cohort.

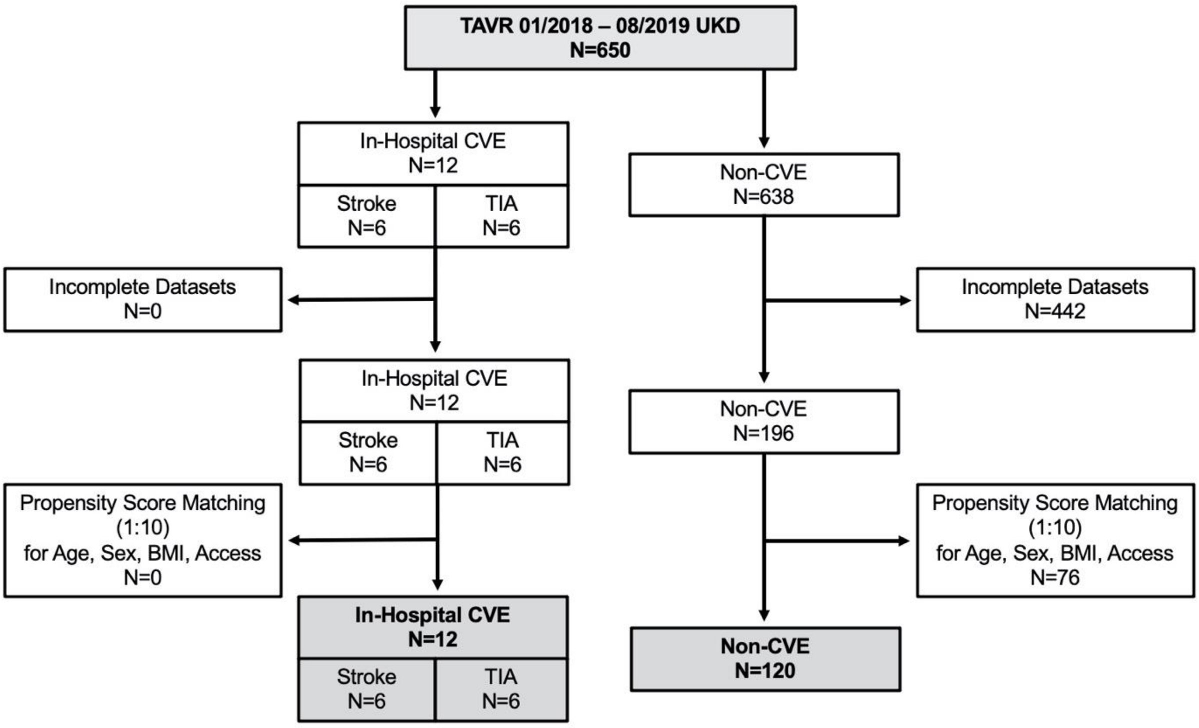

BMI = Body mass index, CVE = Cerebrovascular event, TAVR = Transcatheter aortic valve replacement, TIA = Transient ischemic attack.

Table S1. Patients' baseline characteristics of the derivation cohort.

	All patients (n=577)	Non-CVE group (n=521)	CVE group (n=56)	*p*-Value
Age (years), Mean ± SD	82.2 ± 5.2	82.1 ± 5.2	82.6 ± 4.8	0.469
Female, n (%)	325 (56.3)	292 (56.0)	33 (58.9)	0.679
BMI (kg/m[2]), Mean ± SD	26.9 ± 5.1	26.9 ± 5.1	26.8 ± 4.9	0.896
NYHA class III/IV, n (%)	442 (76.6)	400 (76.8)	42 (75.0)	0.766
CCS class III/IV, n (%)	97 (16.8)	87 (16.7)	10 (17.9)	0.826
Diabetes mellitus, n (%)	145 (25.1)	132 (25.3)	13 (23.2)	0.728
COPD, n (%)	173 (30.0)	158 (30.3)	15 (26.8)	0.583
Arterial hypertension, n (%)	549 (95.1)	496 (95.2)	53 (94.6)	0.853
Atrial fibrillation, n (%)	188 (32.6)	169 (32.4)	19 (33.9)	0.821
Pulmonary hypertension, n (%)	406 (70.4)	367 (70.4)	39 (69.6)	0.901
CAD, n (%)	430 (74.5)	391 (75.0)	39 (69.6)	0.378
PAD, n (%)	183 (31.7)	163 (31.3)	20 (35.7)	0.499
CVD, n (%)	105 (18.2)	91 (17.5)	14 (25.0)	0.165
Prior CVE, n (%)	55 (9.9)	45 (8.6)	10 (17.9)	0.026*
GFR (<50 mL/min), n (%)	247 (42.8)	221 (42.4)	26 (46.4)	0.564
EuroSCORE II (%), Mean ± SD	8.2 ± 8.5	8.2 ± 8.3	8.9 ± 10.5	0.557
STS score (%), Mean ± SD	7.6 ± 6.9	7.6 ± 7.1	7.6 ± 5.2	0.935
HR (log.ES ≥20%), n (%)	332 (57.5)	294 (56.4)	38 (67.9)	0.100
HAS-BLED Score, Mean ± SD	3.5 ± 0.9	3.5 ± 0.9	3.7 ± 0.9	0.048*
CHA_2DS_2VASc Score, Mean ± SD	4.8 ± 1.2	4.8 ± 1.2	5.2 ± 1.1	0.009**
OAC, n (%)	167 (28.9)	155 (29.8)	12 (21.4)	0.218
NOAC, n (%)	56 (9.7)	49 (9.4)	7 (12.5)	0.429
SAPT, n (%)	221 (38.3)	200 (38.4)	21 (37.5)	0.976
DAPT, n (%)	99 (17.2)	88 (16.9)	11 (19.6)	0.561
LVEF (%), Mean ± SD	50.0 ± 13.9	50.3 ± 13.9	47.1 ± 14.3	0.421
PAsys (>50mmHg), n (%)	51 (8.8)	49 (9.4)	2 (3.6)	0.250
Hospital stay (days), Mean ± SD	11.6 ± 8.1	10.9 ± 6.8	18.7 ± 14.1	<0.001***

Values are mean ± SD or n (%). Parameters are shown with *p*-values (**p*<0.05, ***p*<0.01, ****p*<0.001). BMI = Body mass index, CAD = Coronary artery disease, CCS = Canadian Cardiovascular Society, COPD = Chronic obstructive pulmonary disease, CVD = Cerebrovascular disease, CVE = Cerebrovascular event, DAPT = Dual antiplatelet therapy, GFR = Glomerular filtration rate, HR = High risk, LVEF = Left ventricular ejection fraction, NOAC = New oral anticoagulation, NYHA = New York

Heart Association, OAC = Oral anticoagulation, PAD = Peripheral vascular disease, PAsys = Systolic pulmonal artery pressure, SAPT = Single antiplatelet therapy, STS = Society of Thoracic Surgeons.

Table S2. Patients' characteristics of the validation cohort.

	**All patients (n=132)**	**Non-CVE group (n=120)**	**CVE group (n=12)**	***p*-Value**
Age (years), Mean ± SD	79.4 ± 6.7	79.5 ± 6.6	77.6 ± 8.2	0.340
Female, n (%)	68 (51.5)	62 (51.7)	6 (50.0)	0.912
BMI (kg/m²), Mean ± SD	26.5 ± 4.8	26.5 ± 4.9	27.0 ± 4.1	0.739
NYHA class III/IV, n (%)	108 (81.8)	99 (82.5)	9 (75.0)	0.521
CCS class III/IV, n (%)	6 (4.5)	5 (4.2)	1 (8.3)	0.509
Diabetes mellitus, n (%)	34 (25.8)	30 (25.0)	4 (33.3)	0.529
COPD, n (%)	41 (31.1)	37 (30.8)	4 (33.3)	0.858
Arterial hypertension, n (%)	118 (89.4)	109 (90.8)	9 (75.0)	0.089
Atrial fibrillation, n (%)	50 (37.9)	46 (38.3)	4 (33.3)	0.734
Pulmonary hypertension, n (%)	71 (53.8)	64 (53.3)	7 (58.3)	0.740
CAD, n (%)	96 (72.7)	89 (74.2)	7 (58.3)	0.240
PAD, n (%)	30 (22.7)	27 (22.5)	3 (25.0)	0.844
CVD, n (%)	21 (15.9)	17 (14.2)	4 (33.3)	0.083
Prior CVE, n (%)	22 (16.7)	20 (16.7)	2 (16.7)	1.000
GFR (<50 mL/min), n (%)	47 (35.6)	40 (33.3)	7 (58.3)	0.085
EuroSCORE II (%), Mean ± SD	5.4 ± 5.1	5.2 ± 4.9	7.3 ± 6.8	0.178
STS score (%), Mean ± SD	4.5 ± 3.1	4.5 ± 2.9	5.0 ± 5.4	0.600
HR (log.ES ≥20%), n (%)	58 (43.9)	53 (44.2)	5 (41.7)	0.868
HAS-BLED Score, Mean ± SD	2.7 ± 0.9	2.7 ± 0.9	2.5 ± 0.8	0.484
CHA_2DS_2VASc Score, Mean ± SD	4.7 ± 1.3	4.7 ± 1.4	4.3 ± 1.1	0.373
OAC, n (%)	21 (15.9)	18 (15.0)	3 (25.0)	0.367
NOAC, n (%)	34 (25.8)	32 (26.7)	2 (16.7)	0.450
SAPT, n (%)	39 (29.5)	34 (28.3)	5 (41.7)	0.334
DAPT, n (%)	32 (24.2)	28 (23.3)	4 (33.3)	0.441
LVEF (%), Mean ± SD	53.4 ± 12.9	53.7 ± 13.0	50.8 ± 12.4	0.460
PAsys (>50mmHg), n (%)	13 (9.8)	11 (9.2)	2 (16.7)	0.695
Hospital stay (days), Mean ± SD	10.0 ± 7.4	9.5 ± 6.3	15.8 ± 13.9	0.147

Values are mean ± SD or n (%). Parameters are shown with *p*-values. BMI = Body mass index, CAD = Coronary artery disease, CCS = Canadian Cardiovascular Society, COPD = Chronic obstructive pulmonary disease, CVD = Cerebrovascular disease, CVE = Cerebrovascular event, DAPT = Dual antiplatelet therapy, GFR = Glomerular filtration rate, HR = High risk, LVEF = Left ventricular ejection fraction, NOAC = New oral anticoagulation, NYHA = New York Heart Association, OAC = Oral anticoagulation, PAD = Peripheral vascular disease, PAsys = Systolic pulmonal artery pressure, SAPT = Single antiplatelet therapy, STS = Society of Thoracic Surgeons.

Table S3. Patients' characteristics of the derivation cohort and the validation cohort.

	All patients (n=709)	Derivation cohort (n=577)	Validation cohort (n=132)	*p*-Value
Age (years), Mean ± SD	81.7 ± 5.5	82.2 ± 5.2	79.4 ± 6.7	<0.001***
Female, n (%)	393 (55.4)	325 (56.3)	68 (51.5)	0.316
BMI (kg/m^2), Mean ± SD	26.8 ± 5.0	26.9 ± 5.1	26.5 ± 4.8	0.410
NYHA class III/IV, n (%)	550 (77.6)	442 (76.6)	108 (81.8)	0.195
CCS class III/IV, n (%)	103 (14.5)	97 (16.8)	6 (4.5)	<0.001***
Diabetes mellitus, n (%)	179 (25.2)	145 (25.1)	34 (25.8)	0.881
COPD, n (%)	214 (30.2)	173 (30.0)	41 (31.1)	0.808
Arterial hypertension, n (%)	667 (94.1)	549 (95.1)	118 (89.4)	0.012*
Atrial fibrillation, n (%)	238 (33.6)	188 (32.6)	50 (37.9)	0.245
Pulmonary hypertension, n (%)	477 (67.3)	406 (70.4)	71 (53.8)	<0.001***
CAD, n (%)	526 (74.2)	430 (74.5)	96 (72.7)	0.671
PAD, n (%)	213 (30.0)	183 (31.7)	30 (22.7)	0.042*
CVD, n (%)	126 (17.8)	105 (18.2)	21 (15.9)	0.535
Prior CVE, n (%)	77 (10.9)	55 (9.9)	22 (16.7)	0.018*
GFR (<50 mL/min), n (%)	294 (41.5)	247 (42.8)	47 (35.6)	0.130
EuroSCORE II (%), Mean ± SD	7.9 ± 7.9	8.2 ± 8.5	5.4 ± 5.1	<0.001***
STS score (%), Mean ± SD	7.0 ± 6.2	7.6 ± 6.9	4.5 ± 3.1	<0.001***
HR (log.ES ≥20%), n (%)	390 (55.0)	332 (57.5)	58 (43.9)	0.005**
HAS-BLED Score, Mean ± SD	3.4 ± 0.9	3.5 ± 0.9	2.7 ± 0.9	<0.001***
CHA_2DS_2VASc Score, Mean ± SD	4.8 ± 1.2	4.8 ± 1.2	4.7 ± 1.3	0.400
OAC, n (%)	188 (26.5)	167 (28.9)	21 (15.9)	0.002**
NOAC, n (%)	90 (12.7)	56 (9.7)	34 (25.8)	<0.001***
SAPT, n (%)	260 (36.7)	221 (38.3)	39 (29.5)	0.060
DAPT, n (%)	131 (18.5)	99 (17.2)	32 (24.2)	0.059
LVEF (%), Mean ± SD	50.6 ± 13.7	50.0 ± 13.9	53.4 ± 12.9	0.010*

PAsys (>50mmHg), n (%)	64 (9.0)	51 (8.8)	13 (9.8)	0.715
Hospital stay (days), Mean ± SD	11.3 ± 8.0	11.6 ± 8.1	10.0 ± 7.4	0.040*

Values are mean ± SD or n (%). Parameters are shown with *p*-values (*$p<0.05$, **$p<0.01$, ***$p<0.001$). BMI = Body mass index, CAD = Coronary artery disease, CCS = Canadian Cardiovascular Society, COPD = Chronic obstructive pulmonary disease, CVD = Cerebrovascular disease, CVE = Cerebrovascular event, DAPT = Dual antiplatelet therapy, GFR = Glomerular filtration rate, HR = High risk, LVEF = Left ventricular ejection fraction, NOAC = New oral anticoagulation, NYHA = New York Heart Association, OAC = Oral anticoagulation, PAD = Peripheral vascular disease, PAsys = Systolic pulmonal artery pressure, SAPT = Single antiplatelet therapy, STS = Society of Thoracic Surgeons.
